# Reduced trabecular bone mineral density and cortical thickness accompanied by increased outer bone circumference in metacarpal bone of rheumatoid arthritis patients: a cross-sectional study

**DOI:** 10.1186/ar3056

**Published:** 2010-06-21

**Authors:** Daniel Aeberli, Prisca Eser, Harald Bonel, Jolanda Widmer, Gion Caliezi, Pierre-Alain Varisco, Burkhard Möller, Peter M Villiger

**Affiliations:** 1 Department of Rheumatology and Clinical Immunology/Allergology, University Hospital Berne, Freiburgstrasse 18, Bern 3010, Switzerland; 2 Department of Radiology, University Hospital Berne, Freiburgstrasse 18, Bern 3010, Switzerland

## Abstract

**Introduction:**

The objective of this study was to assess three-dimensional bone geometry and density at the epiphysis and shaft of the third meta-carpal bone of rheumatoid arthritis (RA) patients in comparison to healthy controls with the novel method of peripheral quantitative computed tomography (pQCT).

**Methods:**

PQCT scans were performed in 50 female RA patients and 100 healthy female controls at the distal epiphyses and shafts of the third metacarpal bone, the radius and the tibia. Reproducibility was determined by coefficient of varia-tion. Bone densitometric and geometric parameters were compared between the two groups and correlated to disease characteristics.

**Results:**

Reproducibility of different pQCT parameters was between 0.7% and 2.5%. RA patients had 12% to 19% lower trabecular bone mineral density (BMD) (*P *≤ 0.001) at the distal epiphyses of radius, tibia and metacarpal bone. At the shafts of these bones RA patients had 7% to 16% thinner cortices (*P *≤ 0.03). Total cross-sectional area (CSA) at the metacarpal bone shaft of pa-tients was larger (between 5% and 7%, *P *< 0.02), and relative cortical area was reduced by 13%. Erosiveness by Ratingen score correlated negatively with tra-becular and total BMD at the epiphyses and shaft cortical thickness of all measured bones (*P *< 0.04).

**Conclusions:**

Reduced trabecular BMD and thinner cortices at peripheral bones, and a greater bone shaft diameter at the metacarpal bone suggest RA spe-cific bone alterations. The proposed pQCT protocol is reliable and allows measuring juxta-articular trabecular BMD and shaft geometry at the metacarpal bone.

## Introduction

Juxta-articular bone loss is one of the earliest radiographic findings of active rheuma-toid arthritis (RA) [1,2]. Recently, loss of bone mass at the metacarpal shafts meas-ured on plain radiographs of the hand has been found to be predictive of subsequent joint damage in patients with active rheumatoid arthritis [1,3]. So far, reduced bone mass at the metacarpal bone shaft in RA has been documented in a number of stud-ies using Digital X-ray Radiogrammetry (DXR) [1,3-5] or at the hand by Dual X-ray Absorptiometry (DXA) [3,6-8]. Trabecular bone loss in RA patients, however, has on-ly been studied at the iliac crest [9] and at the distal radius [10-12], where it was found to be lower in RA patients than in controls [9,11].

Peripheral Quantitative Computed Tomography (pQCT) is a three-dimensional measuring technique that allows the assessment of cross-sectional bone geometry and volumetric bone mineral density (vBMD). In contrast to two-dimensional methods like DXA and DXR, pQCT allows the determination of bone geometry of bone cross-section independent of bone size. To date, no study has examined vBMD and cross-sectional bone geometry of the metacarpal bones in RA. We have recently used pQCT for measuring metacarpal bone in patients with diffuse idiopathic skeletal hy-perostosis (DISH) patients [13]. Interestingly, juvenile idiopathic arthritis measure-ments of bone mineral density and geometry by pQCT have shown that articular and periarticular inflammation is associated with bone loss and changes in bone geome-try, in particular reduced cortical thickness and increased bone cross-sectional area [14-17].

The aim of the present study was to assess vBMD and bone geometry of metacarpal bone, radius and tibia in patients with established RA by pQCT and to compare these peripheral bone parameters to those of healthy controls.

## Materials and methods

We conducted a prospective observational study comparing female RA patients to a control group. The study protocol was approved by the Ethics Committee of the Can-ton of Bern.

### Subjects

Consecutive female RA patients, fulfilling the American College of Rheumatology cri-teria [18], seen in the Department of Rheumatology and Clinical Immunology, Insel-spital Bern, were included. For the control group, we recruited healthy female volun-teers by locally distributed flyers and advertisement on the hospital internal web. In-clusion criteria were for both groups age 20 to 90 years. Exclusion criteria for both groups were bone metabolic diseases, hyper-/hypoparathyroidism, hy-per/hypothyreoidism, chronic renal insufficiency, cancer, pregnancy, lactation and drug addiction on the basis of medical history and questionnaires for osteoporosis risk factors. For the control group, established osteoporosis and previous or present bisphosphonate therapy were also exclusion criteria. All patients and volunteers gave written informed consent.

### Assessment of disease characteristics

Erosiveness was assessed by total Ratingen score [19] for the non-dominant hand by a study-independent radiologist and a rheumatologist. From medical records, most recently determined Rheumatoid Factor (RF) and anti-Cyclic Citrullinated Peptide an-tibody (anti-CCP), disease duration, modified disease activity score (DAS)including 28 joints [20], therapy with regard to anti-tumor necrosis factor (anti-TNF), bisphos-phonate and glucocorticoids were extracted.

### Bone measurements

Measurements were performed with a Stratec XCT 3000 scanner (Stratec Medizin-technik, Pforzheim, Germany). This pQCT apparatus measures attenuation of x-rays which are linearly transformed into hydroxylapatit (HA) densities. Unlike some other pQCT scanners, the Stratec XCT 3000 is calibrated with respect to water which is set at 60 mg HA, so that fat results in 0 mg HA [21]. HA equivalent densities are auto-matically calculated from the attenuation coefficients by employing the manufac-turer's phantom which itself is calibrated with respect to the European Forearm Phan-tom (Erlangen, Germany) [21]. PQCT measurements of the radius and the metacar-pal bone were performed on the non-dominant side and at the tibia on the opposite leg.

#### Metacarpal measurements

**Figure 1:**
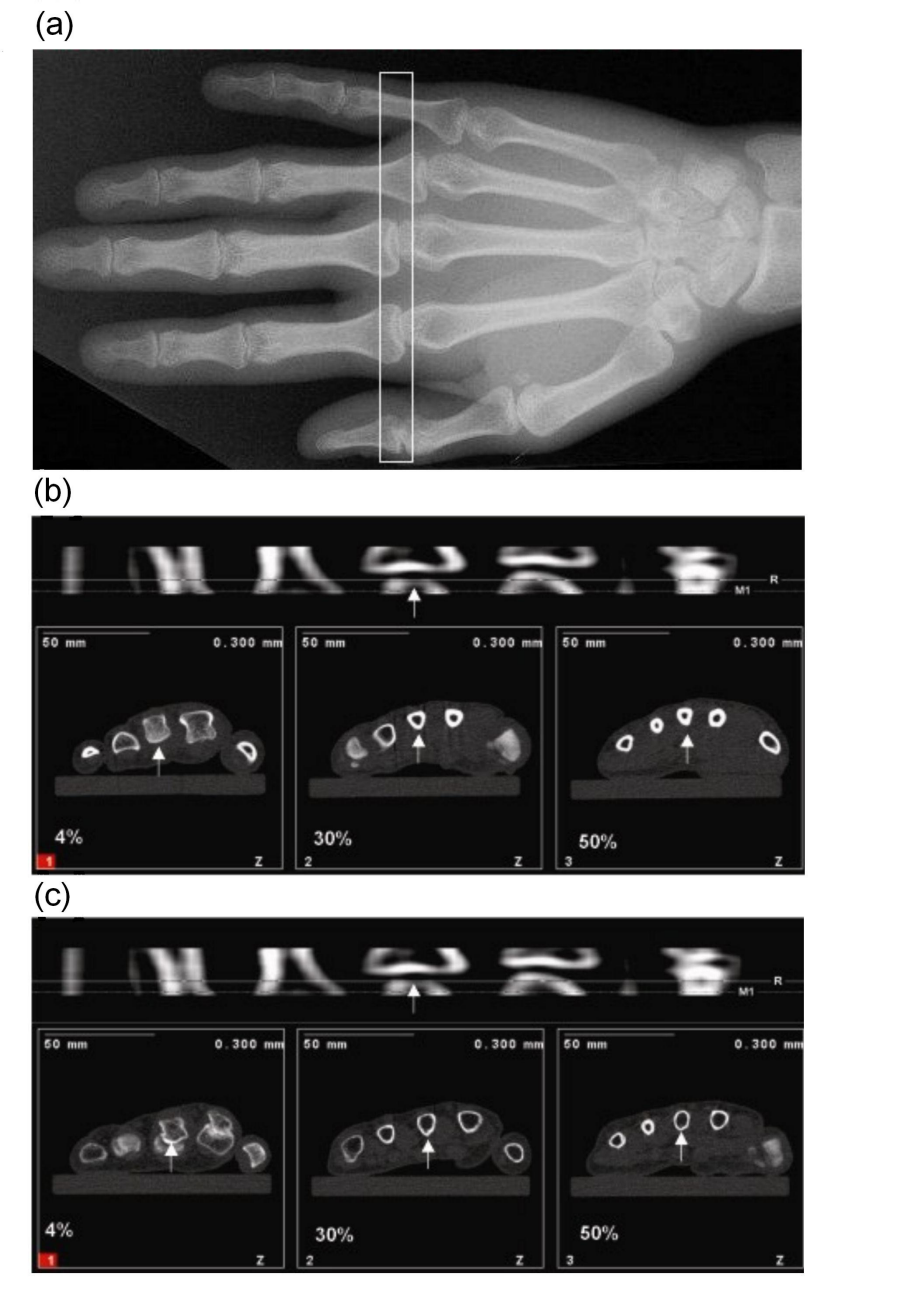
**Placement of scout view (a) and typical scout view with reference line placement and the 3 metacarpal scans in a healthy reference par-ticipant (b) and RA patient (c)**. The third metacarpal bone is indicated with a white arrow.

Length of metacarpal bone III of the non-dominant hand was palpated and measured from base to head by measuring tape to the nearest 5 mm. The subjects were seated in a chair side on to the gantry and had their arm and hand resting on a custom made flat wooden holder. The arm was abducted to 90 degrees with the elbow, wrist and fingers extended and palm facing down. Several Velcro straps centered the middle finger and arm on the slightly padded wooden holder and held the arm securely in place. The Velcro strap around the middle finger attached along the middle axis of the wooden holder ensured that the axis of the third metacarpal bone was in line with the central axis of the forearm and perpendicular to the gantry. A scout view was per-formed of the head of ossa metacarpalia III (Figure 1a) and the reference line was placed at the distal end of the bone (Figure 1b, c). Scans were performed at 4%, 30% and 50% of the total bone length measured from the distal bone end. Slice thickness was 2.2 mm, voxel size was set at 0.3 mm edge length, and scanning speed was set at 15 mm/s. Reference line placement and typical pQCT images of metacarpal measurements of a control subject and an RA patient are illustrated in Figure 1b, c.

#### Radius and tibia measurements

Radius bone length was set equal to ulnar length, which was measured to the near-est 5 mm with a measuring tape by palpation from the olecranon to the ulnar styloid. Tibia length was determined from the medial knee joint cleft to the end of the medial malleolus. A scout view of the distal end of the tibia/radius was performed and the automated detection algorithm provided by the manufacturer was used to place the reference line at the distal bone end. Scans were performed according to manufac-turer's recommendations at 4% and 66% of the bone's total length measured from the reference line. Slice thickness was 2.2 mm, and voxel size was set at 0.5 mm with a scanning speed of 20 mm/s. The manufacturer's software XCT 6.00 B (Stratec Medizintechnik, Pforzheim, Germany) was used for analysis.

#### Measuring parameters

Epiphyseal scan (4%): The periosteal surface of each bone's epiphysis was found by a contour algorithm based on thresholding at 200 mg/cm^3 ^(metacar-pals) and 180 mg/cm^3 ^(radius and tibia, contour mode 1 and peel mode 1 of the software). Bone mineral content (BMC) per cm slice thickness, total cross-sectional area (CSA) and total volumetric bone mineral density (BMD) were de-termined. Concentric pixel layers were then peeled off from the bone's perimeter until a central area covering 50% (metacarpals) or 45% (radius and tibia) of the total bone CSA was left. Trabecular BMD was determined from this central area.

Diaphyseal scans (30% and 50% for metacarpals, 66% for radius and tibia): The threshold for the periosteal surface was set at 280 mg/cm^3 ^and from this BMC and total CSA were calculated. Cortical bone was selected by thresholding at 710 mg/cm^3 ^(contour mode 1 and peel mode 1), and from this, corti-cal CSA and cortical BMD were calculated. Cortical thickness was calculated based on the assumption that the bone shaft be cylindrical from total CSA, which included the bone marrow, and cortical CSA of the diaphyseal scans. At the 50% scan of the metacarpal bone the relative cortical area was calculated as cortical CSA/total CSA. This relative cortical area is proportional to the metacarpal index commonly meas-ured on standard x-rays or digitised radiography. Muscle CSA was determined by se-lecting the area with a lower threshold of 40 mg/cm^3 ^and an upper threshold of 280 mg/cm^3 ^HA density after smoothing the image (con-tour mode 3 and peel mode 1, and contour mode 1 and peel mode 2 for subtracting the bone area).

### Precision of metacarpal bone measurements

Nine subjects of the control group volunteered to have a total of four measurements of metacarpal bone III of the same hand within a maximal time span of three weeks (or three months in one subject). The two operators who performed the measure-ments of this study completed two measurements each in each of the nine subjects. If repeat measurements were performed on the same day, subjects were completely repositioned between the two scans.

### Data analysis

To determine reproducibility of the new protocol coefficients of variation (CV) for met-acarpal bone measurements were calculated as root-mean-square (RMS) averages of standard deviations [22] including all four measurements of all nine subjects. Nine-ty-five percent confidence intervals (CI) of the CVs were calculated by bootstrapping (n = 2,000 simulations). Because some of the bone parameters were not normally distributed, Mann-Whitney tests were performed between the reference group and the RA group with regard to age, height, weight, and muscle CSA of the forearm and lower leg. Mann-Whitney tests were also performed for all bone parameters of the third metacarpal bone, the radius and the tibia. For easier interpretation of the results, means and standard deviations of all parameters for each group as well as relative differences between groups were calculated. Furthermore, ANCOVAs with muscle CSA as covariate (forearm muscle CSA for radial and metacarpal bone parameters and lower leg muscle CSA for tibial bone parameters) were performed for all bone parameters to adjust for the significant between-group differences in lower leg mus-cle CSA and trend for forearm muscle CSA. In the RA group Spearman correlation coefficients were calculated between trabecular and total BMD, as well as cortical thickness and total Ratingen score. Statistical analyses were performed with SPSS version 17.0 (SPSS Inc., Zurich, Switzerland), and statistical significance was set at an alpha of 0.05.

## Results

### Subject parameters

A total of 50 RA patients and 100 control subjects fulfilled the selection criteria and were recruited for the present study. Two patients had metal implants at the non-dominant radius, in these patients the dominant radius was measured. Subject char-acteristics are presented in Table 1. The two groups were comparable with regard to age and weight. However, RA patients had a 9% smaller muscle CSA at the lower leg (*P *= 0.01) and muscle CSA at the lower arm of the RA group tended to be 5% smaller (*P *= 0.10). The RA patients' height tended to be 10% small-er (*P *= 0.09).

**Table 1:** Subject anthropometric data (mean ± standard deviation)

Parameter	RA patients	Reference group	*P*-value
Number of subjects	50	100	
Age (y)	55.3 ± 11.4	54.1 ± 12.9	0.481
Height (cm)	163.4 ± 6.2	165.0 ± 5.7	0.092
Weight (kg)	67.0 ± 13.8	63.6 ± 9.8	0.183
Forearm muscle CSA (cm^2^)	24.0 ± 4.0	25.3 ± 3.5	0.102
Lower leg muscle CSA (cm^2^)	58.1 ± 11.3	63.7 ± 10.6	**0.014**

*P*-values are indicated for two-sided Mann-Whitney-tests (significant *P*-values in bold). CSA stands for cross-sectional area.

### Clinical parameters

Mean (SD) disease duration was 11.4 (9.5) yrs (median 8.1 yrs) and mean disease activity (DAS28) 4.2 (1.1). Sixty-nine percent were classified erosive, 67% were posi-tive for RF and 85% for anti-CCP. Anti-TNF therapy was previously given to 62% (mean duration was 14.4 months), and bisphosphonates to 35%. Seventy-two per-cent had been on glucocorticoid therapy during the year previous to pQCT measure-ment.

### Precision of metacarpal bone measurements

Coefficients of variation (CV) with 95% CI reflecting the measuring errors for the measured bone parameters at the third metacarpal bone are shown in Table 2. CVs were smaller than or equal to 2.5% for all measured parameters. Upper limits of 95% CI were between 0.99% and 2.99%.

**Table 2:** Results of pQCT reproducibility measurements (four measurements in each of nine subjects) of the third metacarpal bone

Scan location	Bone parameter	Overall mean	SD	CV (%)	95% CI of CV (%)
Distal epiphysis (4%)	BMC (mg/mm)	46.4	0.76	1.64	1.18 to 2.14
	Total CSA (mm^2^)	124.1	2.82	2.27	1.72 to 2.77
	Total BMD (mg/cm^3^)	373.6	3.69	0.99	0.77 to 1.24
	Trabecular BMD [mg/cm^3^]	331.0	8.10	2.45	1.96 to 2.99
Shaft (30%)	BMC (mg/mm)	52.4	0.71	1.35	0.98 to 1.70
	Total CSA (mm^2^)	75.7	1.42	1.87	1.32 to 2.26
	Cortical CSA (mm^2^)	38.1	0.59	1.55	1.13 to 2.10
	Cortical BMD (mg/cm^3^)	1,166.38	10.29	0.88	0.62 to 1.12
Shaft (50%)	BMC (mg/mm)	52.8	0.44	0.84	0.62 to 1.11
	Total CSA (mm^2^)	58.8	0.86	1.46	1.01 to 1.92
	Cortical CSA (mm^2^)	39.3	0.40	1.02	0.67 to 1.33
	Cortical BMD (mg/cm^3^)	1,205.8	8.51	0.71	0.41 to 0.99

Indicated are overall mean value, standard deviation (SD), Coefficient of variation (CV) and 95% confidence interval (CI) of the CV. BMC stands for bone mineral content per mm of slice thickness, CSA for cross-sectional area and BMD for volumetric bone mineral density.

### Bone characteristics in RA patients

**Figure 2:**
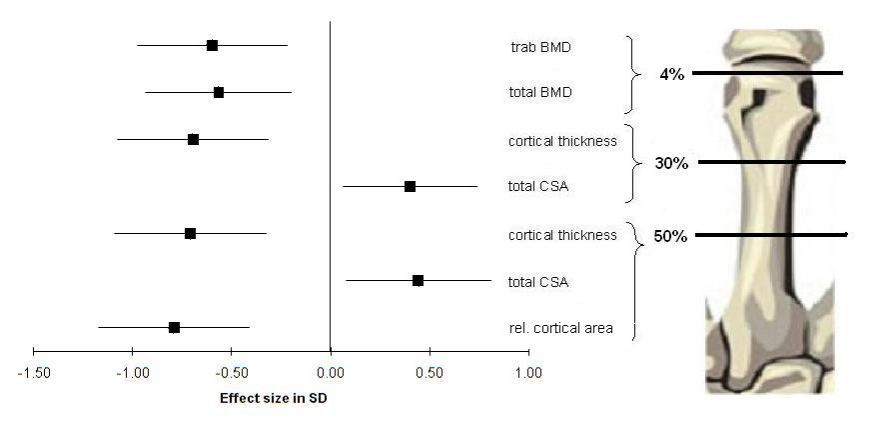
**Effect size of bone parameters at the metacarpal bone between RA patients and healthy controls**. The error bars indicate 95% confidence interval of the between group differences in mean SD of both groups.

Trabecular BMD at the distal epiphyses of metacarpals, radius and tibia were 13% to 19% lower in the RA group compared to the control group (*P *≤ 0.001, Table 3). Total BMD was 10% lower at the distal tibia and 9% lower at the distal third meta-carpal bone in the RA group (*P *≤ 0.001). Cortical thickness was 7% to 16% thinner at all three shafts (*P *< 0.03). Total CSA was between 5% and 7% greater at the 30% and 50% site of the metacarpal shaft in the RA groups (*P *< 0.02). Cortical BMD was smaller in the RA group (except for the tibial shaft), a finding most probably caused by partial volume effect [23] due to the thinner cortices rather than real differences. The relative cortical area was 12.5% smaller in the RA patients (*P *= 0.001). Differences in standard deviations of metacarpal bone parameters between the RA and control group are shown in Figure 2.

**Table 3:** Bone parameters at the radius, tibia, and third metacarpal bone in RA patients and controls (means ± sd), P-values of two-sided Mann-Whitney tests (significant values are in bold), and difference between mean values of RA and control group

		Group	RA (n = 50)		Ref (n = 100)		Mann-Whitney test *P*-value	Relative difference [%]
Bone	Site	Parameter	Mean	SD	Mean	SD		
Radius	4%	BMC (g/cm)	1.03	0.23	1.09	0.17	0.087	-5.5
		Total CSA (mm^2^)	339.3	55.3	338.4	48.2	0.599	0.3
		Total BMD (mg/cm^3^)	305.3	58.2	325.5	53.1	0.076	-6.2
		Trab. BMD (mg/cm^3^)	151.6	47.3	186.1	38.4	**0.000**	-18.5
	66%	BMC (g/cm)	0.89	0.21	0.98	0.16	**0.009**	-9.2
		Total CSA (mm^2^)	139.4	26.1	132.6	20.7	0.110	5.1
		Cort. CSA (mm^2^)	63.3	19.6	71.8	13.3	**0.017**	-11.8
		Cort. BMD (mg/cm^3^)	1,094.0	78.7	1137.6	57.3	**0.000**	-3.8
		Cort. Thickness (mm)	1.80	0.59	2.14	0.45	**0.001**	-15.9
Tibia	4%	BMC (g/cm)	2.76	0.60	3.02	0.43	**0.002**	-8.6
		Total CSA (mm^2^)	1,087.6	161.2	1071.7	126.9	0.251	1.5
		Total BMD (mg/cm^3^)	255.0	47.7	284.6	43.7	**0.000**	-10.4
		Trab. BMD (mg/cm^3^)	192.5	43.5	222.9	37.3	**0.000**	-13.6
	66%	BMC (g/cm)	3.54	0.54	3.72	0.43	0.085	-4.8
		Total CSA (mm^2^)	549.9	81.4	548.3	67.6	0.956	0.3
		Cort. CSA [mm^2^)	266.4	43.9	282.6	33.4	**0.048**	-5.7
		Cort. BMD (mg/cm^3^)	1,117.0	54.8	1130.6	36.6	0.152	-1.2
		Cort. Thickness (mm)	3.78	0.65	4.05	0.52	**0.029**	-6.7
MCP3	4%	BMC (mg/mm)	40.48	9.96	43.70	7.18	**0.031**	-7.4
		Total CSA (mm^2^)	124.8	19.6	124.2	13.0	0.751	0.5
		Total BMD (mg/cm^3^)	321.6	66.0	351.8	44.4	**0.001**	-8.6
		Trab. BMD (mg/cm^3^)	266.0	80.5	303.2	44.9	**0.001**	-12.3
	30%	BMC (mg/mm)	43.68	11.34	47.07	6.16	0.130	-7.2
		Total CSA (mm^2^)	76.10	13.60	71.26	11.52	**0.015**	6.8
		Cort. CSA (mm^2^)	31.59	8.49	34.66	4.22	0.088	-8.9
		Cort. BMD (mg/cm^3^)	1,100.1	87.5	1146.2	57.7	**0.005**	-4.0
		Cort. Thickness (mm)	1.19	0.36	1.38	0.23	**0.005**	-13.8
	50%	BMC (mg/mm)	44.97	11.39	48.25	6.22	0.102	-6.8
		Total CSA (mm^2^)	59.69	8.15	56.66	6.13	**0.019**	5.3
		Cort. CSA (mm^2^)	33.04	8.56	36.06	4.70	**0.043**	- 8.4
		Cort. BMD (mg/cm^3^)	1,153.0	76.5	1,189.9	44.7	**0.008**	-3.1
		Cort. Thickness (mm)	1.48	0.45	1.71	0.28	**0.001**	-13.5
		relative cortical area	0.56	0.14	0.64	0.08	**0.001**	-12.5

*P*-values are rounded to three decimal places, values of 0.000 are equivalent to *P *< 0.0005. BMC stands for bone mineral content per mm of slice thickness, CSA for cross-sectional area, BMD for volumetric bone mineral density, trab for trabecular and cort for cortical.

Results of the ANCOVAs adjusting for muscle CSA are shown in Table 4. Differences in intercepts of the two groups (assessed at mean muscle CSA) remained significant for trabecular BMD and cortical thickness of all three bones (except cortical thickness of the tibia), and total CSA of the metacarpal bone was even more significantly great-er in the RA group after adjustment for muscle CSA. In addition, many of the per-formed ANCOVAs showed a significant interaction between group and muscle CSA (difference in slopes on Table 4), meaning that the slope of the linear relationship be-tween muscle and bone parameter was different in the two groups. All bone parame-ters of the RA group, except cortical BMD, were associated with muscle CSA (slope of RA group in Table 4).

**Table 4:** Results of Analyses of covariance with factor RA - group - status and covariate muscle cross - sectional area (CSA)

Bone	Site	Parameter	Difference between intercepts (*P*-value)		Difference between slopes (*P-*value)		Slope of RA group (*P*-value)	
**Radius**	**4%**	**BMC (g/cm)**	0.01	(0.610)	** - 0.01**	**(0.045)**	**0.04**	**(0.000)**
		Total CSA (mm^2^)	- 10.64	(0.190)	- 2.10	(0.319)	7.62	**(0.000)**
		Total BMD (mg/cm^3^)	15.94	(0.099)	- 1.76	(0.483)	4.16	**(0.035)**
		Trab. BMD (mg/cm^3^)	28.50	**(0.000)**	- 3.61	(0.052)	5.29	**(0.000)**
	66%	BMC (g/cm)	0.05	**(0.050)**	- 0.01	(0.154)	0.03	**(0.000)**
		Total CSA (mm^2^)	- 10.82	**(0.003)**	- 1.54	(0.108)	3.84	**(0.000)**
		Cort. CSA (mm^2^)	5.57	**(0.023)**	- 0.69	(0.281)	2.70	**(0.000)**
		Cort. BMD (mg/cm^3^)	42.58	**(0.000)**	0.55	(0.869)	1.07	(0.686)
		Cort. Thickness (mm)	0.29	**(0.001)**	- 0.01	(0.657)	0.05	**(0.004)**
Tibia	4%	BMC (g/cm)	0.08	(0.267)	- 0.02	**(0.003)**	0.04	**(0.000)**
		Total CSA (mm^2^)	- 53.01	**(0.031)**	- 5.18	**(0.017)**	7.80	**(0.000)**
		Total BMD (mg/cm^3^)	22.13	**(0.008)**	- 0.52	(0.471)	1.66	**(0.005)**
		Trab. BMD (mg/cm^3^)	21.71	**(0.003)**	- 1.68	**(0.008)**	2.15	**(0.000)**
	66%	BMC (g/cm)	0.02	(0.742)	- 0.01	**(0.024)**	0.04	**(0.000)**
		Total CSA (mm^2^)	- 17.41	(0.178)	- 0.91	(0.424)	3.13	**(0.001)**
		Cort. CSA (mm^2^)	2.75	(0.619)	- 1.38	**(0.005)**	2.98	**(0.000)**
		Cort. BMD (mg/cm^3^)	11.42	(0.169)	0.05	(0.944)	0.38	(0.518)
		Cort. Thickness (mm)	1.14	(0.171)	- 0.02	**(0.038)**	0.03	**(0.000)**
MCP3	4%	BMC (mg/cm)	1.40	(0.266)	- 0.35	(0.287)	1.41	**(0.000)**
		Total CSA (mm^2^)	- 2.74	(0.299)	- 0.42	(0.543)	1.71	**(0.002)**
		Total BMD (mg/cm^3^)	21.60	**(0.014)**	- 3.36	(0.138)	7.52	**(0.000)**
		Trab. BMD (mg/cm^3^)	25.98	**(0.008)**	- 4.28	(0.092)	8.64	**(0.000)**
	30%	BMC (mg/cm)	1.67	(0.194)	- 0.60	(0.074)	1.47	**(0.000)**
		Total CSA (mm^2^)	- 7.33	**(0.000)**	- 0.86	(0.092)	2.08	**(0.000)**
		Cort. CSA (mm^2^)	1.76	(0.056)	- 0.49	**(0.039)**	1.13	**(0.000)**
		Cort. BMD (mg/cm^3^)	43.98	**(0.001)**	- 1.39	(0.667)	2.61	(0.300)
		Cort. Thickness (mm)	0.16	**(0.001)**	- 0.01	(0.419)	0.03	**(0.014)**
	50%	BMC (mg/cm)	1.45	(0.229)	- 0.43	(0.168)	1.58	**(0.000)**
		Total CSA (mm^2^)	- 4.40	**(0.000)**	- 0.44	(0.113)	1.18	**(0.000)**
		Cort. CSA (mm^2^)	1.60	(0.073)	- 0.38	(0.099)	1.25	**(0.000)**
		Cort. BMD (mg/cm^3^)	32.25	**(0.002)**	- 1.97	(0.452)	4.51	**(0.029)**
		Cort. Thickness (mm)	0.18	**(0.002)**	- 0.01	(0.588)	0.04	**(0.000)**
		relative cortical area	0.07	**(0.000)**	- 0.00	(0.617)	0.01	**(0.017)**

For bone parameters of the radius and metacarpal bone muscle CSA of the forearm (cm^2^) was used, and for the tibia muscle CSA at the lower leg (cm^2^) was used. Bold P-values indicate significant coefficients. P-values are rounded to three decimal places, values of 0.000 are equivalent to P < 0.0005. BMC stands for bone mineral content per mm of slice thickness, BMD for volumetric bone mineral density, trab for trabecular and cort for cortical.

### Relationship between erosive status and bone parameters

Total Ratingen score correlated negatively with total and trabecular BMD at all three measured bone sites (r between -0.36 and - 0.48, *P *≤ 0.011), and with corti-cal thickness at all three measured shafts (radius and tibia: r between - 0.31 and - 0.38, *P *≤ 0.04, metacarpal shaft: r between - 0.42 and to - 0.51, *P *≤ 0.003).

## Discussion

The detailed three-dimensional assessment of peripheral bone vBMD and geometry of the present study shows a systemically lower trabecular BMD and thinner cortices in RA patients and a localised greater outer bone shaft circumference at the meta-carpal bone.

Trabecular BMD at the third metacarpal bone, the radius and the tibia was lower in RA patients than controls. This was in accordance with earlier studies using DXA where the RA population was found to have lower total BMD at the distal metacarpal bone [7], at the distal radius [10,12,24], and the hip [11,25,26].

The metacarpal bone shafts of our RA patients were thinner and had a greater outer bone diameter (Figure 2). These results are in good agreement with a recent publica-tion on patients with polyarticular juvenile idiopathic arthritis [14]. The between-group deficits in trabecular BMD could not be accounted for by adjustment to muscle cross-sectional area (CSA), indicating that the bone deficit in RA patients was greater than what would be expected as a result of their atrophied muscles. The same was true for cortical thickness of the radius and metacarpal shafts. However, at the tibia shaft, differences in cortical thickness disappeared after adjusting for muscle CSA (Table 4). It should be noted that the slope of the muscle CSA to cortical thickness relation-ship differed, indicating that the thinning of the tibial cortex with decreasing muscle CSA was amplified in the RA group. In addition, the greater outer metacarpal diame-ter in our RA patients stands in contrast to the smaller muscle CSA. This may indi-cate that while part of the deficit in trabecular BMD and cortical thickness may have been caused by muscle atrophy, other disease related processes further reduced jux-ta-articular trabecular BMD and altered shaft geometry. Two pathomechanisms for decreased cortical thickness and increased outer circumference of the shaft are cur-rently discussed: First, bony apposition is seen as a compensatory mechanism to counterbalance inflammation to induced cortical thinning [14]. Second, periosteal bone formation is seen as a repair process in inflammation-induced increased bone turnover [27,28]. Irrespective of the causality of the greater outer bone shaft diame-ter, the result is an improved bone resistance against bending and torsion [29].

We found a significant negative correlation between erosive score and total and tra-becular BMD as well as cortical shaft thickness at all measured bones. This is in ac-cordance with the relationship between development of erosions at the wrist and fin-gers and the loss of areal BMD at the metacarpal bone measured by digital x-ray ra-diogrammetry [1,4,30]. Significantly lower baseline areal BMD at the hip [31,32] and spine [33] was found in early RA patients with erosive development, pointing to a general bone loss as consequence of a systemic inflammatory process. Our data of the radius and tibia support the notion of a systemic inflammatory process. Our more detailed analysis of vBMD and bone geometry showed lower trabecular vBMD at the radius and tibia and a thinner shaft cortical thickness at the radius independent of muscle atrophy, suggesting that systemic inflammatory processes may be involved. However, the greater shaft outer diameter was seen only at the metacarpal bone shaft suggesting RA-specific alterations at the metacarpal bone.

The presented data document a good performance of a newly developed protocol for measuring volumetric BMD and bone geometry by pQCT at the third metacarpal bone. Reproducibility was similar to previous studies measuring metacarpal areal BMD in RA patients by DXA [7,34] and in studies using pQCT (XCT 3000) at the ra-dius [35], tibia [35-37], femur [35-37] and humerus [35]. CVs at the metacarpal mid-shaft (50% scan) of our protocol ranged from 0.7% to 1.5%. This is higher than the CV of 0.14% to 0.3% reported for digital X-ray radiogrammetry [38], and most proba-bly due to the higher susceptibility to malpositioning. We have also performed inter- and intra-operator Intraclass Correlation Coefficients (ICC) and have found all ICCs > 0.85. Indeed, most ICCs were > 0.99, and they were similar between and within the two operators, indicating that the measuring protocol was not operator-sensitive.

A limitation of the present study is the large number of conducted statistical tests. Therefore, even *P*-values well below 0.05 should be interpreted carefully. However, the main results of this study, namely the between-group differences in tra-becular BMD and cortical thickness of all measured bones had *P*-values of ≤ 0.005 (except for the tibia shaft cortical thickness with a *P*-value of 0.03), and total CSA of the metacarpal bone had a *P*-value of < 0.02. Further, RA patients were on various medications that influence bone metabolism (biologicals, glucocorticoids, bisphosphonates). However, the aim of the present study was to compare a cohort of RA patients treated according to current common practice with healthy controls. Our results highlight that despite the bone protective effects of bio-logicals and bisphosphonates, trabecular BMD and cortical thickness were reduced at all measured skeletal sites in RA patients. While there were no clear associations between bone parameters and use of biological or glucocorticoids, patients on bisphosphonates had significantly lower trabecular BMD at all measured epiphyses (with diagnosis of osteoporosis being the treatment indication)(data not shown).

## Conclusions

In RA patients, trabecular BMD at the distal epiphyses of metacarpals, radius and tib-ia was lower compared to controls, and cortical thickness was thinner at the shafts. Furthermore, the outer bone diameter at the metacarpal shaft was larger in RA pa-tients compared to controls. This suggests inflammation- and probably disease- spe-cific mechanisms being operative in bone remodelling. It remains to be shown whether these changes may help to monitor disease progression and guide treat-ment intensity.

## Abbreviations

ANCOVA: analysis of covariance; anti-CCP: anti-Cyclic Citrullinated Peptide antibody; anti-TNF: anti-tumor necrosis factor; BMC: bone mineral content; BMD: bone mineral density; CI: confidence inter-val; CSA: cross-sectional area; CV: coefficient of variation; DAS: disease activity score; DXA: dual x-ray absorptiometry; DXR: digital x-ray radiogrammetry; HA: hy-droxylapatite; ICC: Intraclass Correlation Coefficients; pQCT: peripheral quantitative computed tomography; RA: rheumatoid arthritis; RF: rheumatoid factor; RMS: root-mean-square; vBMD: volumetric bone mineral density.

## Competing interests

The authors declare that they have no competing interests.

## Authors' contributions

DA was involved in the conception and design, acquisition of data, analysis and in-terpretation of data, writing and critical revision of the manuscript, final approval of the version to be published, and acquisition of funding. PE was involved in the conception and design, acquisition of data, analysis and interpretation of data, writing and critical revision of the manuscript, and final approval of the version to be published. HB was involved in the acquisition of data, critical revision of the manu-script, and final approval of the version to be published. JW, GC, PAV and BM were involved in the acquisition of data, critical revision of the manuscript, and final approval of the version to be published. PV was involved in the conception and design, critical revision of the manuscript, final approval of the version to be published, and acquisition of funding.
